# Development of Eight Wireless Automated Cages System with Two Lickometers Each for Rodents

**DOI:** 10.1523/ENEURO.0526-21.2022

**Published:** 2022-08-03

**Authors:** Mariana Cardoso Melo, Paulo Eduardo Alves, Marianna Nogueira Cecyn, Paula Mendonça C. Eduardo, Karina P. Abrahao

**Affiliations:** Departamento de Psicobiologia, Escola Paulista de Medicina, Universidade Federal de São Paulo (Unifesp), São Paulo, Brazil

**Keywords:** behavior, drinking microstructure, lickometer, mice, prototype validation, two-bottle choice

## Abstract

Drinking behavior has been used in fundamental research to study metabolism, motivation, decision-making and different aspects of health problems, such as anhedonia and alcohol use disorders. In the majority of studies, liquid intake is measured by weighing the bottles before and after the experiment. This method does not tell much about the drinking microstructure, e.g., licking bouts and periods of preference for each liquid, which could be valuable to understand drinking behavior. To improve data acquisition of drinking microstructure, companies have developed lickometer devices that acquire timestamps when animals approach or drink from a specific sipper. Nevertheless, commercially available devices have elevated costs. Here, we present a low-cost alternative for a lickometer system that allows wireless data acquisition of licks from eight cages with two sippers each. We ran a three-phase validation protocol to ensure (1) proper choice of the sensor to detect licks; (2) adaptation of the device to a wireless transmission and realistic *in silico* tests; and (3) *in vivo* validation to test the correlation between the amount of licks measured by the lickometer and the bottle weight. The capacitive sensor presented appropriate recall and precision for our device. After adaptation to wireless transmission, the *in silico* validation demonstrated low reading and transmission errors even when tested in extreme simultaneous licking conditions. Finally, we observed a positive correlation between water or ethanol consumption and lick count, showing that the lickometers can be used for *in vivo* studies interested in rodent drinking microstructure.

## Significance Statement

This study presents an innovative and low-cost solution for drinking behavioral studies: a lickometer system based on an open-source hardware platform with a user-friendly interface software, capable of simultaneously receiving data from eight automated cages with two drinking bottles each. The lickometer brings an accessible device to acquire high-quality and detailed data. This device also has the possibility to be adaptable to new types of sensors or other neuroscience tools capable of measuring brain activity simultaneously to the behavior.

## Introduction

Liquid intake is an essential animal behavior. Together with feeding and sexual behavior, drinking water (or other liquids) is influenced by genetic and environmental factors (Ramirez and Sprott, 1978; Bachmanov et al., 2002; Bainier et al., 2017). Scientists have been using drinking behavior in rodents to study metabolism, motivation, decision-making, and different aspects of psychological and medical problems, such as alcohol use disorders (Jeanblanc et al., 2019). Sometimes, animals are exposed to more than one drinking bottle containing different liquids and have to decide what to drink. This protocol has often been called the two-bottle choice model (Barkley-Levenson and Crabbe, 2015; Panksepp et al., 2017). In these studies, the volume consumed is recorded usually by weighing bottles before and after animals drink from them.

Although these studies have produced valuable data for different fields of psychobiology, there is greater complexity in drinking behavior. Drinking is highly influenced by light/dark circadian rhythms (Eisenhardt et al., 2015; Bainier et al., 2017; Gamsby et al., 2017), indicating that measures of drinking in different periods can reveal different results. In addition, different drinking patterns can produce interesting interpretations of the data, even when the total volume consumed is similar. In humans, for example, researchers have studied the microstructure of sucrose intake to detect different motivational states (Gero et al., 2019). Differences in drinking initiation, bout duration, and timing may indicate compulsive-like states in rodents, as specific drinking behavioral characteristics are observed in ethanol aversion-resistant intake protocol (Darevsky et al., 2019; Darevsky and Hopf, 2020). Thus, collecting detailed data is an important step for further evaluation of this type of behavior.

In order to study drinking microstructure, scientists have been using automated lickometers (Barkley-Levenson and Crabbe, 2015; Cains et al., 2017; Frie and Khokhar, 2019; Godynyuk et al., 2019). These devices can collect timestamps every time animals approach or drink from a specific sipper. However, many commercially available lickometers are extremely costly for laboratories. Alternatively, low-cost and open-source devices have been found in the literature, created with electronic code prototyping platforms (Longley et al., 2017; Frie and Khokhar, 2019; Godynyuk et al., 2019). There are at least two types of these devices: photoelectric/barrier sensor-based (Frie and Khokhar, 2019) or capacitive sensor-based (Longley et al., 2017). In both cases they are designed to have one circuit board for two bottles on each animal cage. If the behavioral experiment requires a significant number of animals, specific cages for each rodent could imply great development efforts. Furthermore, most of the validation procedures use only correlation analysis between the animal’s sipper interactions and bottle weight or preference for two types of liquids, such as water and sucrose (Longley et al., 2017; Godynyuk et al., 2019).

The present work focused on developing an innovative and low-cost solution for drinking behavioral studies where it is necessary to run groups of animals at the same time. We propose a system capable of simultaneously receiving data from eight automated cages with two drinking bottles each, using a wireless system. Our lickometer device enhances the experiment’s robustness by collecting synchronized information from rodent behavior experiments. To reach the final system, we present in this paper three development and validation steps as the following: (1) selection of the proper licking detection sensor; (2) translation to wireless transmission and validation with emulated signals; and (3) *in vivo* validation using mice drinking in two-bottle lickometer cages.

## Materials and Methods

### Selection of the proper licking detection method

#### System description

**Figure 1.**
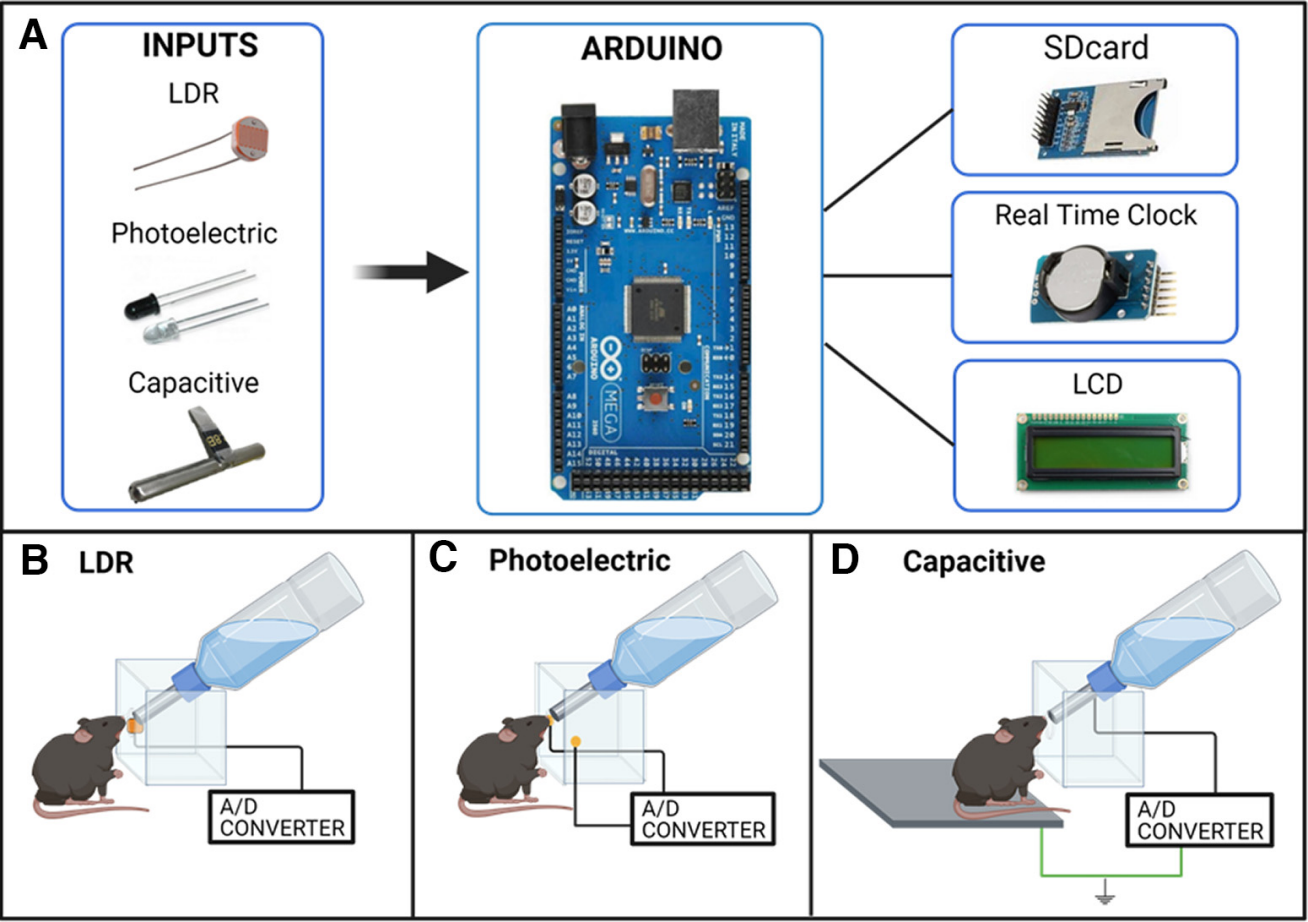
Schematic illustration of prototypes. ***A***, Electronic components, where the three selected sensors are shown in the INPUTS box: LDR, photograph emitter/photosensor (photoelectric), and the capacitive sensor (with a 65-mm stainless steel nozzle). An Arduino MEGA was used as an analog-digital signal converter. The shields are shown on the right: an SD card module to record the collected data, a real-time clock module (DS3231) and an LCD 16 × 2 to show, licks counting in real time. ***B–D***, Prototypes’ schematic showing the lick cabin for each sensor, the mouse positioning, the liquid bottle and the sensors. ***B***, LDR prototype sensor was positioned at one side of the lick cabin. ***C***, The photoelectric prototype sensor had the photo emitter aligned to the photoreceiver on the lick cabin. ***D***, The capacitive prototype sensor was created by the connection between the stainless-steel nozzle to an analog input of the Arduino (black wire), and the platform was connected to the ground (Gnd, green wire). Created with BioRender. For electronic schematic diagram, please see Extended Data Figure 1-1.

We developed three prototypes with the respective types of sensors: light-dependent resistor (LDR), photoelectric, and capacitive (touch), as shown in Figure 1*A* (electronic schematic diagram of the three prototypes is presented in Extended Data Fig. 1-1). All prototypes consisted of an Arduino MEGA, used for the analog/digital conversion (A/D converter) of the sensor’s signal, a Secure Digital (SD) card module to store the data, a real-time clock module (DS3231) to precisely time the licks events and a Liquid Crystal Display (LCD) 16 × 2 to show the number of licks and date/time of occurrence.

10.1523/ENEURO.0526-21.2022.f1-1 Extended DATAFigure 1-1 Extended figure shows the electronic schematic diagram of the three built prototypes: (***A***) photoelectric, (***B***) LDR, and (***C***) capacitive sensor. Download Figure 1-1, TIF file.

The licking apparatus was adapted according to the specificity of each sensor (Fig. 1*B–D*). One hole aligned to the bottle nozzle was made for the LDR sensor positioning (Fig. 1*B*). If the mice approached the cabin, the change in the luminosity altered the resistance of the LDR and, consequently, its voltage. The LDR prototype counted a new lick when a voltage threshold was reached. The photoelectric sensor was adjusted in two aligned holes in the opposite walls of the cabin (Fig. 1*C*). One hole corresponded to the photoelectric emitter and the other to the photoreceiver. Thus, in this case, the lick count occurred when the animal interrupted the signal between emitter and receiver. Finally, the capacitive sensor prototype required an adaptation of the house cage to weld a 65-mm stainless steel to the sipper and connect it to the Arduino’s analog input. Also, an aluminum platform was installed in the cage to create the system ground when mice step on top of it (Fig. 1*D*). In order to turn the Arduino pin, connected to the steel bottle nozzle, into a capacitive sensor we used the library *CapacitiveSensor* (Bagder, 2015). A lick was counted when the mice simultaneously touched the bottle nozzle and the aluminum platform.

#### Animals

The animal experiments were approved by the Comissão de Ética em Uso de Animais (CEUA), Universidade Federal de São Paulo (#1482300519). For prototype validation, we used four C57Bl/6 mice (two females and two males; 30–90 days old at the beginning of experiments, from the Centro de Desenvolvimento de Modelos Experimentais para Medicina e Biologia - CEDEME). Animals were individually housed in ventilated racks and kept on a 12/12 h light/dark cycle (light onset at 7 a.m.) with *ad libitum* access to food and water.

#### Experimental procedure

Water and food were withdrawn from the mice’s home cage 2 h before the test in order to increase motivation. We first acquired data from lickometer cages without the presence of a mouse, for 1 min to capture the sensor’s baseline values. Mice were then individually placed in the experimental cage and a bottle with 5% sucralose solution was placed on the licking cabin for each prototype. Researchers observed whether the dimension and position of the drinking bottle were ergonomic for mice. We chose an ergonomic design to avoid mice having any kind of difficulty accessing the drinking sippers, in relation to the height (5 cm of the floor) and angle (60°) of the sipper. Additionally, the licking threshold (value above baseline considered a lick event) was estimated for each prototype by observing the sensor values when the animal approached or moved away from the bottle nozzle. Finally, we designed the initial validation experiment to compare the three prototypes as described below.

All mice were tested twice in each prototype. The experiments were recorded by a camera positioned to record the mouse’s licks behavior and the LCD that exhibited the counts. Each experiment lasted as long as necessary for the prototypes to register at least 100 licks per mouse. The verification of a new lick occurred every 50 ms. A 5-ms delay after a licking event was used to ensure the system did not count duplicate licks whenever a lick detection occurred.

Four researchers watched all the videos. In order to visualize licks, the speed of the videos was slowed down to 0.25×. Researchers detected the presence of true positives, false positives or false negatives, and the time each lick occurred for each video. True positives were reported when an animal licked the sipper and the prototype correctly measured it; false positives, when the system accounted for a new lick but the animal did not do it; false negatives, when the animal did lick the sipper but the system did not count it. Two metrics, precision and recall, were estimated based on these values. Precision corresponds to the ratio of true positives among true and false positives, and recall corresponds to the ratio of true positives in relation to false negatives and true positives. Thus, precision informs the proportion of actual correct positive identification of licks and recall informs the proportion of actual positive lick counts identified correctly. Recall and precision data were compared between prototypes by an independent *t* test (GraphPad Prism), with a level of significance of *p* < 0.05.

### Translation to wireless transmission and *in silico* validation with emulated signals

#### System description

Based on the sensor’s selection with greater precision and recall obtained in the first stage of validation, eight house cages were adapted to have two bottle-coupled sensors.

**Figure 2.**
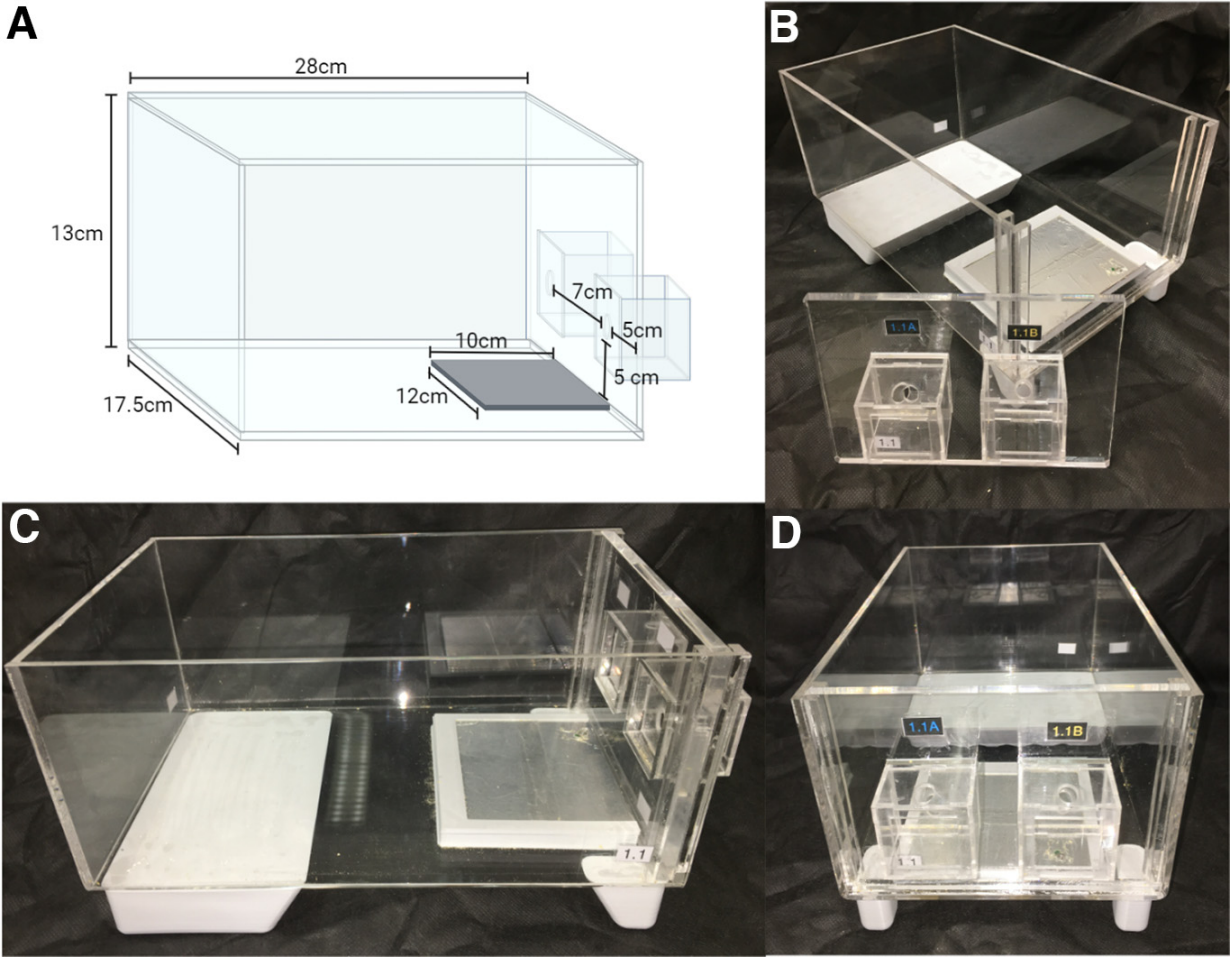
***A***, Designed acrylic cage dimensions for the lickometer. ***B***, Removable wall with the cabins where drinking sippers are positioned. ***C***, Lateral view. ***D***, Front view.

**Figure 3.**
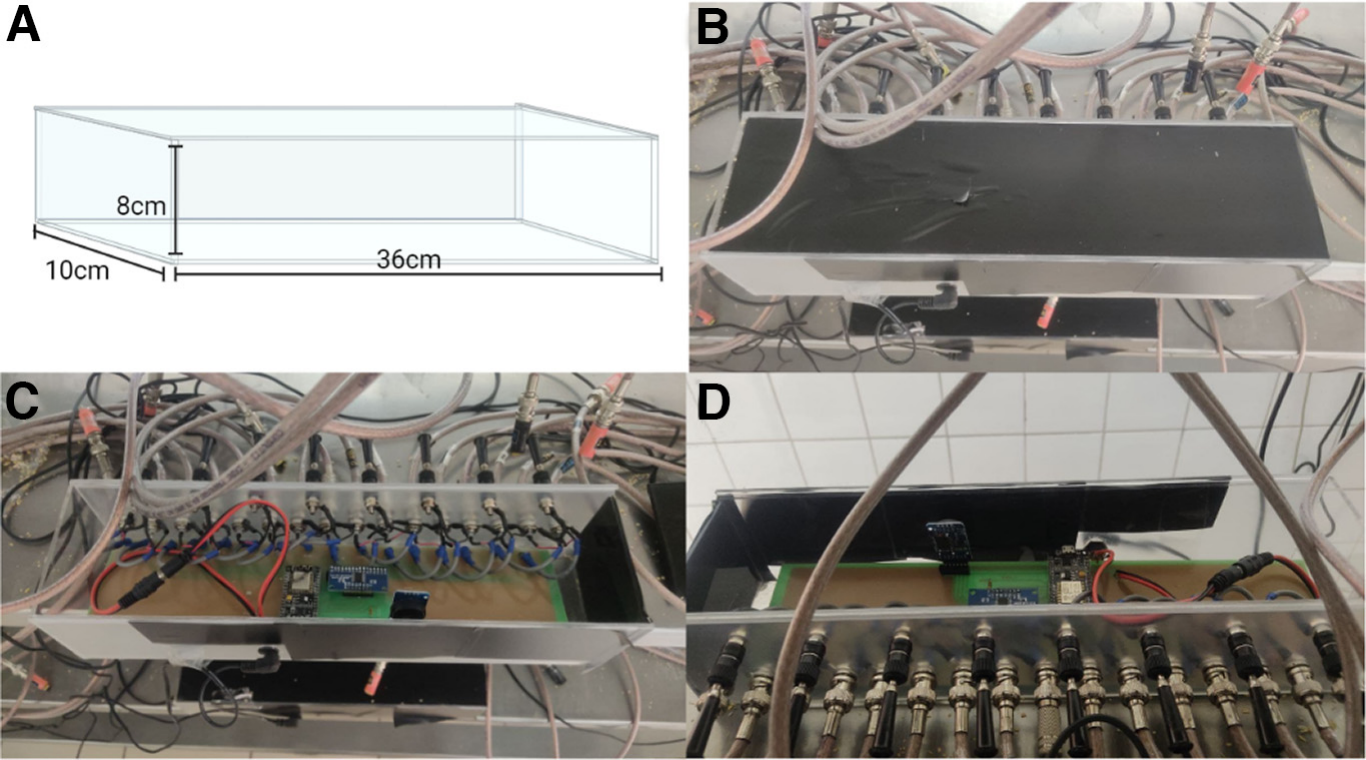
***A***, Acrylic box designed to store the electronic circuit. ***B***, Top view. ***C***, Top view of the open circuit box. ***D***, Front view shows the cables that connect the sensors with the electronic circuit.

Acrylic cages were designed to support two drinking sippers. Its dimensions can be seen in Figure 2*A*. Also, the support for the drinking sippers was planned to be removable (Fig. 2*B*), allowing scientists to remove it in a way to help maintenance and cleaning of the cage. We also developed an electronic circuit to acquire the signals from eight cages, using 95% insulated cables, and send them wirelessly to a desktop computer (Fig. 3). A real-time clock module was used to timestamp the precise time of each lick. As the ESP8266 NodeMCU module has only one analog input, a 16-channel multiplexer was used to receive the corresponding signals from all sensors of the lickometers. Then, the transmission circuit sent all data wirelessly to a computer, with the User Datagram Protocol (UDP), through a NodeMCU ESP8266 module.

In order to acquire the typical lick interval of mice and allow the wireless transmission of the data, we set a sampling rate of 60 ms, which is an appropriate sampling rate for measuring licking behavior. According to Boughter et al. (2007), the average licking rate of a C57Bl/6 mouse is 8.5 licks/s, thus the proposed system acquires 16.67 samples per second which is appropriate to detect lick samples.

We also built a protocol to simulate licks *in silico* which allowed us to verify the efficacy of the proposed wireless system. A potentiometer was used as an on/off switch (“on” or “high-level” means a lick; “off” means no-lick). First, we considered a situation where only one mouse in one lickometer cage would lick one sipper. Next, we increased the number of cages in this condition until a situation in which animals in all eight lickometer cages would lick at the same time. Considering the mean licking rate for mice ranges between 6 and 9 Hz (Boughter et al., 2007; Rossi et al., 2016; Williams et al., 2018), we decided to simulate licks representing low and high-frequency situations. Thus, signals were emitted through the digital ports of the microcontroller ESP8266 at frequencies of 5 and 10 Hz, which allowed a minimum interlick interval (ILI) detection of 200 and 100 ms, respectively. The 5 Hz frequency (ILI = 200 ms) was selected to simulate a typical licking rate, so we could verify how our system responds in ordinary situations. Tests performed at 10 Hz represent a scenario where all eight animals would be licking simultaneously at a higher frequency (ILI = 100 ms).

Table 1 lists the electronic components of one lickometer system and the costs in real (Brazilian currency) and estimated cost in US dollars.

**Table 1:** List of materials used to build the full system of eight house cages and the components of the electronic central unit

Materials	Quantity	Unit cost	Total cost	Estimated total cost (US$)
ESP8266	1	R$ 30.00	R$ 30.00	US$ 6.00
Module RTC	1	R$ 25.00	R$ 25.00	US$ 5.00
Module Mux 74 hc4067	1	R$ 15.00	R$ 15.00	US$ 3.00
Pin bar 1 × 40	6	R$ 1,50	R$ 9.00	US$ 1.80
Switched mode power supply 5V 1A	1	R$ 12.00	R$ 12.00	US$ 2.40
PowerSupply connector/jack P4 female 5.5 x 2.1 mm	1	R$ 0.60	R$ 0.60	US$ 0.12
Female BNC connectors	32	R$ 2.20	R$ 70.40	US$ 14.08
Male BNC connector RG59 75R	32	R$ 4.80	R$ 153.60	US$ 30.72
Coax cable RG59 750HM 96%	16	R$ 3.00	R$ 48.00	US$ 9.60
Banana jack 4 mm	8	R$ 1.30	R$ 10.40	US$ 2.08
Cable 0.25 mm	8	R$ 1.00	R$ 8.00	US$ 1.60
Female pin B67	8	R$ 7.00	R$ 56.00	US$ 11.20
Male pin P22	8	R$ 0.16	R$ 1.28	US$ 0.26
FBU 323101 terminal	16	R$ 0.30	R$ 4.80	US$ 0.96
Resistor 100K	2	R$ 0.05	R$ 0,10	US$ 0.02
General expenses (glue, resin…)	1	R$ 100.00	R$ 100.00	US$ 20.00
Printed circuit board for grounding	8	R$ 30.00	R$ 240.00	US$ 48.00
Printed circuit board for wireless central	1	R$ 48.68	R$ 48.68	US$ 9.74
Acrylic box for circuit	1	R$ 200.00	R$ 200.00	US$ 40.00
Acrylic cage	8	R$ 220.00	R$ 1760.00	US$ 352.00
Welding the nozzle to the connector	16	R$ 20.00	R$ 36.00	US$ 7.20
Total cost			R$ 2828.86	US$ 565.78

The values were considered with the Brazilian currency (real, R$) in February 2021 and converted to the United States dollar. The dollar exchange rate was considered on February 23, 2022.

#### Experimental procedure

**Figure 4.**
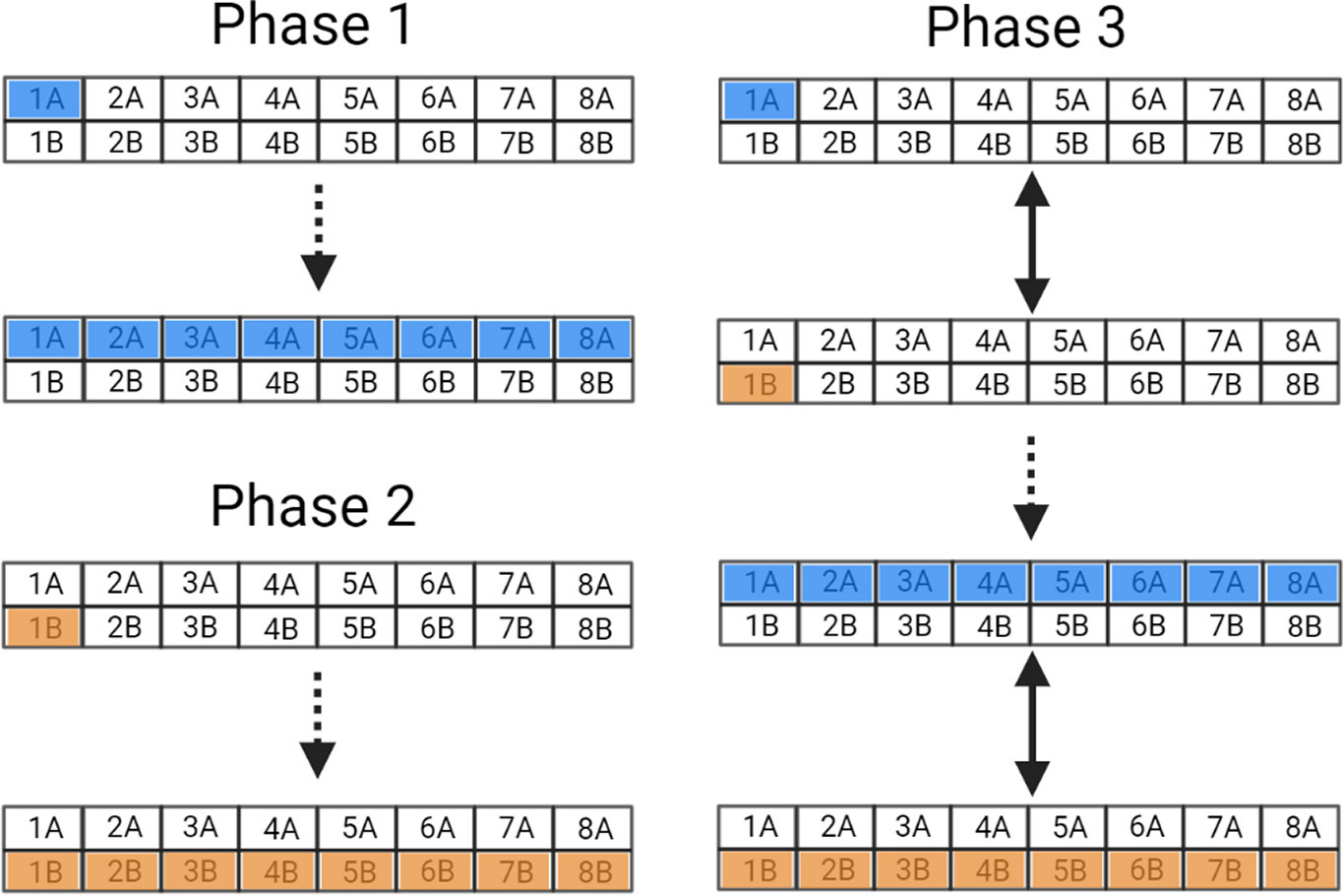
Experimental design of the *in silico* validation protocol with emulated signals. Digital pulses emulating licks were emitted through the digital ports of the microcontroller ESP8266 at frequencies of 5 and 10 Hz. In total, we have built eight cages, each cage has two bottles with sensors that we referred to as A and B. The validation consisted of three phases, in which the number of licks was simulated by alternating each bottle. Phase 1: the pulses initially were sent only from one output A (blue square) to simulate licks from the lickometer A (blue) of one house cage, then two A outputs until simultaneously eight outputs were sending pulses from lickometers A to simulate simultaneous licks from the eight house cages on the lickometers A of each cage. Phase 2: the signals were sent only from the sensor connected to the lickometer B (orange), repeating Phase 1 for this output. Phase 3: pulses were sent from both lickometers A and B, alternately. Thus, signals were sent from one output A (blue) followed by one output B (orange) until simultaneously eight house cages send A and B signals. Created with BioRender.

In total, we have built three sets of eight cages. Each cage had two bottles with sensors that we referred to as A (output A) and B (output B). The *in silico* validation consisted of three phases (Fig. 4). First, emulated lick signals were sent only from output A (Phase 1); then, only from the output B (Phase 2); finally, in the last phase (Phase 3), the signals were sent alternately to each output of the same cage. For each phase, the system sent twelve thousand high-level signals. The number of cages simultaneously receiving the emulated signals increased in each trial. This means that only one sensor of one cage received high-level signals, then only one sensor of two cages received high-level signals simultaneously, and so on, until one sensor of eight cages received simultaneous signals. The same was repeated to the other sensor in the same cages in Phase 2 and alternated for each lickometer in each cage in Phase 3. With the incremental number of cages, it was possible to verify whether the error is correlated with the number of sensors.

We analyzed two types of errors: the reading error (percentage of times the central system did not recognize the licking event correctly before sending it wirelessly to the computer) and the transmission error (the loss of data during transmission via UDP). Both errors were measured for high and low frequency (5 and 10 Hz, respectively). The statistical differences of the reading or transmission errors were compared considering different phases, frequencies and number of cages according to the data normality test. A level of 5% was considered significant, and a Bonferroni *post hoc* was used when necessary.

### *In vivo* validation

#### System description

**Figure 5.**
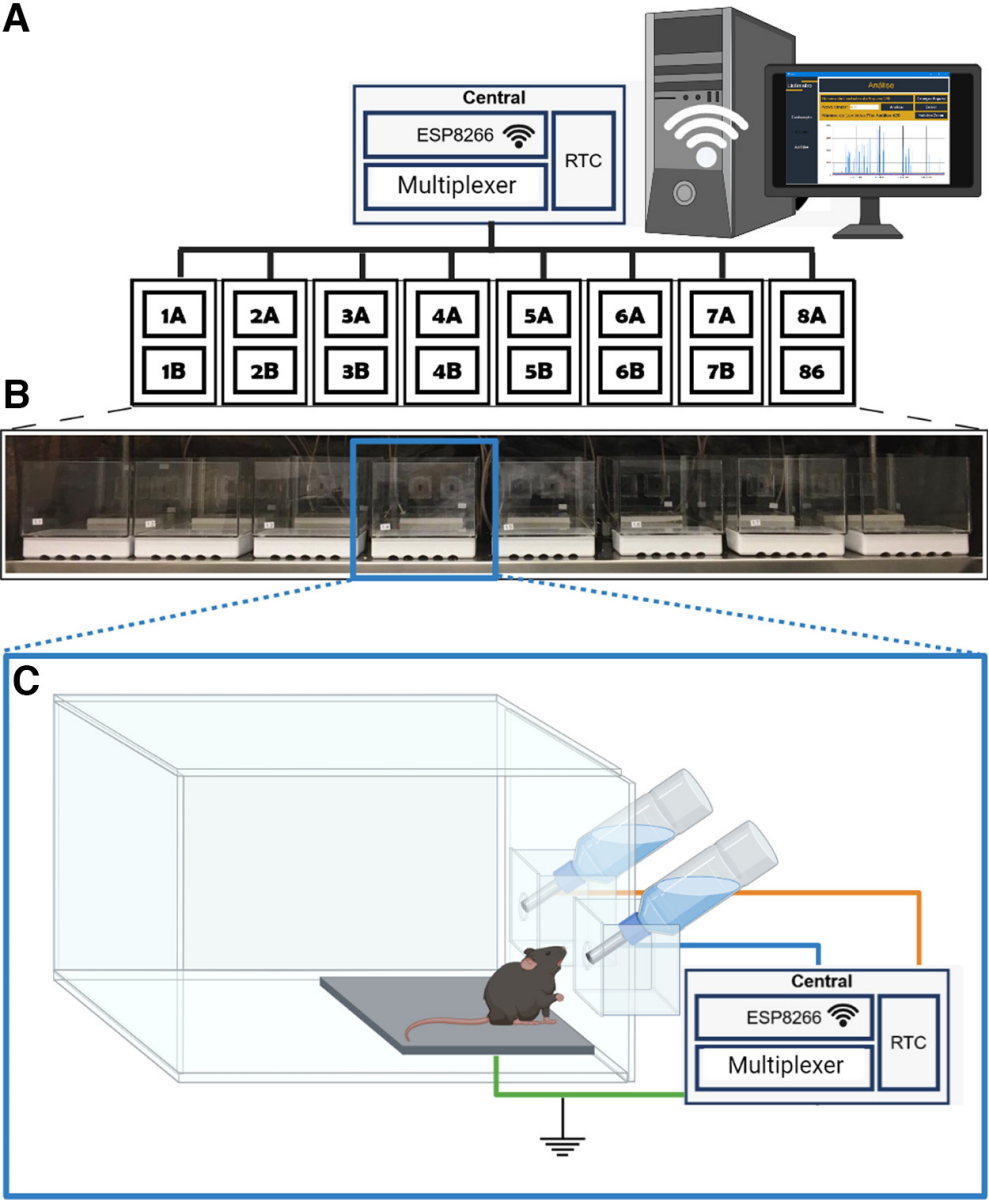
Illustration of one set of lickometer house cages with a detailed view of its parts. ***A***, Schematic of the central system that transmits data from the sensors to the computer wirelessly. The central system consists of an ESP8266, a real-time clock (RTC) and a multiplexer. One central is connected to 16 lickometers from eight boxes. ***B***, One actual set of eight house cages. ***C***, Draft of the final house cage highlighting its parts. Both stainless steel nozzles are connected to an input of the central system (A: lickometer with blue wire and B: lickometer with orange wire), and the platform is connected to the ground (green wire). Created with BioRender.

The last validation step of our system consisted of testing the system with animals individually housed in the lickometer cages to verify the correlation between drinking and licks count registered by the system. We have built three lickometer systems which allowed us to record from 24 cages and 48 lickometers at the same time (one system is shown in Fig. 5). A software in C# was developed to allow acquisition of wireless data and its visualization, including baseline noise and licks as well as to store the sensor raw input data at 16.67 Hz in a text file. Data were then analyzed in MATLAB in which an appropriate licking threshold was established.

#### Animals

The experiments were approved by the CEUA, Unifesp (#5327090819). We used C57Bl/6 mice (24 males and 24 females; seven weeks old at the beginning of experiments, from the CEDEME), housed in two to four animals per home cage in ventilated racks and kept on a 12/12 h light/dark cycle (light onset at 7 a.m.) with *ad libitum* access to food and water.

#### Experimental procedure

In order to observe whether the system could quantify licks that can indicate the amount of liquid drank overnight, one bottle was filled with water and the other was filled with 10% v/v ethanol (C57Bl/6 mice tend to drink high amounts of ethanol). We used an intermittent overnight drinking protocol, in which mice were placed in the lickometer cages 2 h before the lights went off for 16 h, every other night, three nights per week for four weeks. The bottles were weighed before and after each drinking night. We ran two iteration of this experiment with 24 animals in each. Spearman correlations between the total fluid intake (measured by the bottle weights in grams, before and after the overnight test) and the total amount of licks were determined using GraphPad Prism. A level of 0.05 was considered significant.

## Results

### Selection of the proper licking detection method

**Figure 6.**
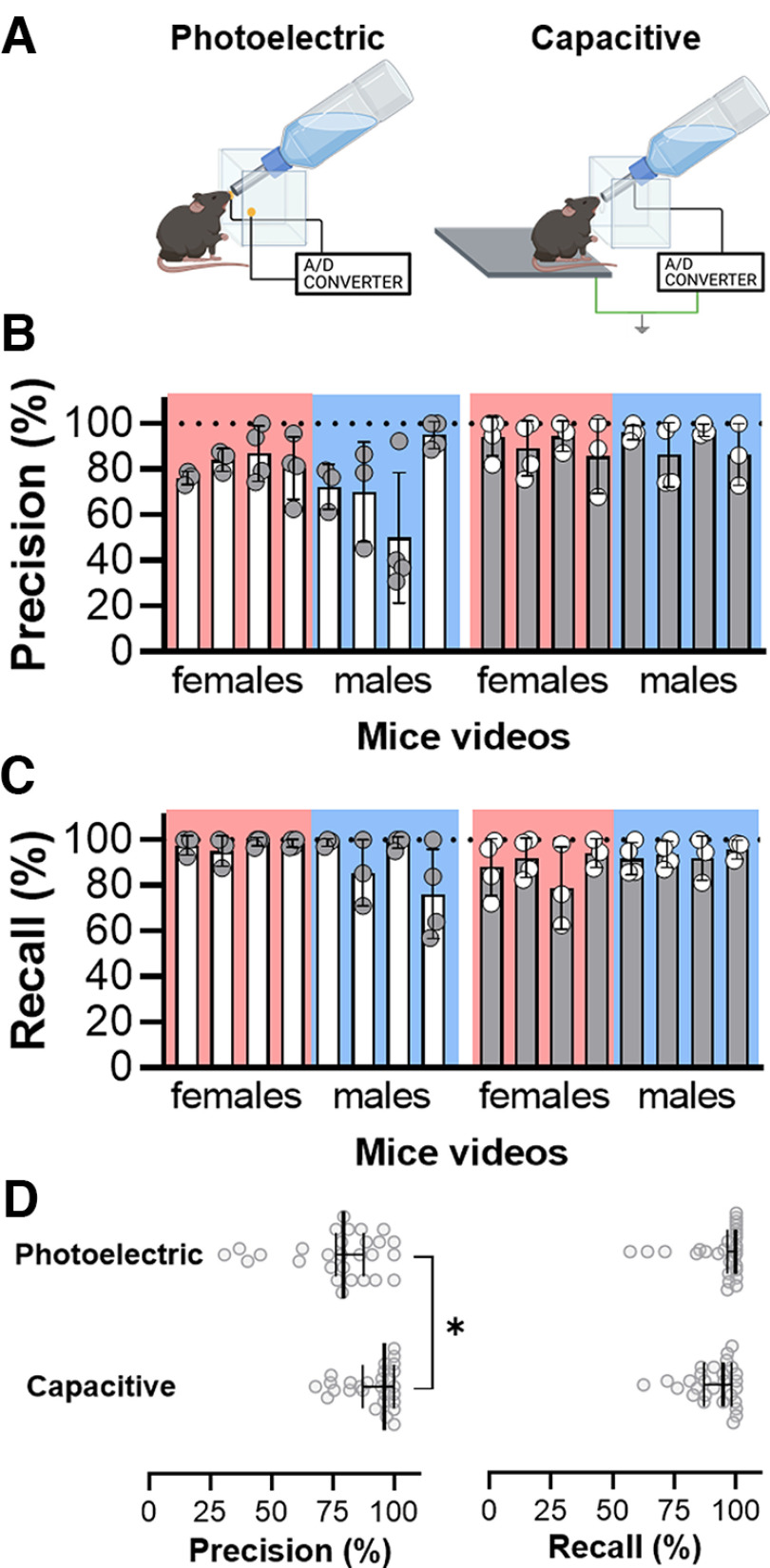
Results of recall and precision values for two prototypes. ***A***, Diagram of the selected prototypes for validation: capacitive and photoelectric sensors connected to the Analog/Digital (A/D) converter. ***B***, Descriptive results of the precision of the photoelectric and capacitive prototypes validation for female and male mice. Each graphical bar is the mean ± standard deviation of the video evaluated by different researchers. ***C***, Descriptive results of the recall of the photoelectric and capacitive prototypes validation for female and male mice. Each graphical bar is the mean ± standard deviation of the video evaluated by different researchers. ***D***, Graphical visualization of results of recall and precision for capacitive and photoelectric prototypes (median ± 95% confidence interval). An independent *t* test detected a significant difference in precision between prototypes (*p < 0.05), being higher with slower dispersion for the capacitive sensor. Recall remained similar for both prototypes, without significant differences.

The LDR prototype was discarded since it did not present adequate stability in the preliminary experiments because of the impact of the environmental luminosity on the data acquisition. We used precision and recall data to compare the capacitive and the photoelectric prototypes (Fig. 6*A*). The capacitive prototype presented significantly higher precision (capacitive: 91.14 ± 5%; photoelectric: 77.02 ± 11.69%; mean ± SD) when compared with the photoelectric prototype (unpaired *t* test; *t*_(54)_ = 3.46; *p* = 0.01; effect size Cohen’s *d* = 0.93; Fig. 6*B*,*D*). The prototypes did not statistically differ for recall (capacitive: 90.79 ± 4.62%; photoelectric: 93.56 ± 8.98%; unpaired *t* test; *t*_(54)_ = 0.98; Fig. 6*C*,*D*). Thus, the capacitive prototype was selected for the next development and validation steps.

### Translation to wireless transmission and *in silico* validation with emulated signals

**Figure 7.**
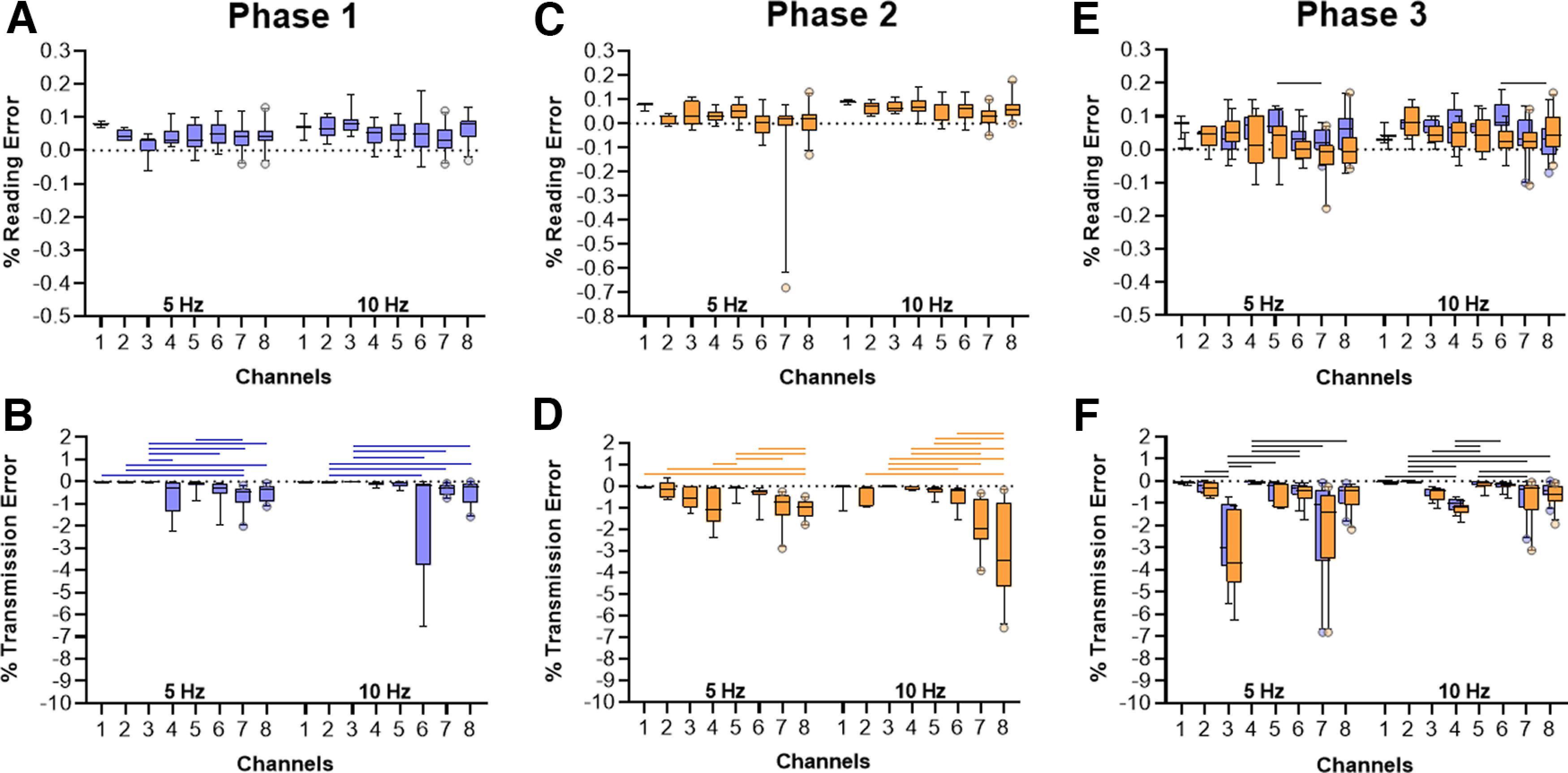
Results of reading and transmission errors obtained with the *in silico* validation of 5 and 10 Hz emulated frequencies for increasing number of cages (channels) receiving the signal (1–8). Box plots represent the mean and the whiskers 9th–95th percentile. Output A is represented in blue and output B is represented in orange. We describe the general range of the errors in this legend using mean ± standard deviation. ***A***, Reading error in Phase 1 (output A) at 5 Hz ranged around 0.0422 ± 0.035%, and at 10 Hz, around 0.0559 ± 0.0424%. ***B***, Reading error in Phase 2 (output B) at 5 Hz ranged around 0.0173 ± 0.0817%, and at 10 Hz, around 0.056 ± 0.0415%. ***C***, Reading error in Phase 3. Considering the 5-Hz frequency, output A shows a reading error ranging around 0.0466 ± 0.0521%, and output B, around 0.0375 ± 0.059%. At 10 Hz, output A shows a reading error ranging around 0.0594 ± 0.054%, and output B, around 0.0679 ± 0.0495%. ***D***, Transmission error in Phase 1 (output A) at 5 Hz ranged around −0.422 ± 0.494%, and at 10 Hz, around −0.484 ± 1.073%. ***E***, Transmission error in Phase 2 (output B) at 5 Hz ranged around −0.661 ± 0.61%, and at 10 Hz, around −1.187 ± 1.618%. ***F***, Transmission error in Phase 3. Considering the 5-Hz frequency, output A shows a transmission error ranging around −0.898 ± 1.312%, and output B, around −1.013 ± 1.405%. At 10 Hz, output A shows a transmission error ranging around −0.441 ± 0.479%, and output B, around −0.537 ± 0.589%. Horizontal lines represent statistically significant differences of each error and each frequency in relation to the amount of channels, calculated by the Bonferroni *post hoc* analysis of an independent-samples Kruskal–Wallis test, *p* < 0.05.

We adapted the initial capacitive prototype and translated the data transmission to a wireless protocol. Three sets of eight cages were built, with two lickometers (capacitive sensors) each. The central unit was used for the *in silico* validation of emulated signals. Figure 7 shows the results of the three simulation phases and their corresponding reading and transmission errors. Since the proposed validation phases in the same frequency required similar hardware capacity, we expected nondivergent transmission and read errors between phases.

Statistical tests were conducted to compare the reading error in different phases at different emulation frequencies considering different output signals (output A, Phase 1 or output B, Phase 2 or both outputs A and B, Phase 3). One independent-samples Kruskal–Wallis test was conducted for all reading error data to compare error among the different phases (H_(5)_ = 60.035; *p* < 0.001; η^2^ = 0.063). The *post hoc* analysis revealed that the reading error (Fig. 7*A–C*) in Phase 1 at 5 Hz was lower than in Phase 3 at 10 Hz (*p* = 0.003). Phase 2 at 5 Hz had a lower reading error than Phase 1 at 10 Hz (*p* < 0.001), Phase 2 at 10 Hz (*p* < 0.001), Phase 3 at 10 Hz (*p* < 0.001) and Phase 3 at 5 Hz (*p* = 0.033). In addition, Phase 3 at 5 Hz had lower reading error than Phase 3 at 10 Hz (*p* < 0.001). Another independent-samples Kruskal–Wallis test was conducted for all transmission error data (H_(5)_ = 45.259; *p* < 0.001; η^2^ = 0.068). The *post hoc* analysis revealed that Phase 1 at 10 Hz had lower transmission error than Phase 2 at 5 Hz (*p* < 0.001) and 10 Hz (*p* < 0.001), as well as than Phase 3 at 5 Hz (*p* < 0.001) and Phase 3 at 10 Hz (*p* = 0.016). Phase 1 at 5 Hz had significantly lower transmission error than Phase 2 at 5 Hz (*p* = 0.015) and than Phase 3 at 5 Hz (*p* = 0.03).

Specific differences within the same frequency and phase among different number of cages (or channels) are represented in Figure 7 and were analyzed by a separate independent-samples Kruskal–Wallis test each. The *post hoc* results are represented in the graph by horizontal lines. No difference in reading error and number of cages was observed in Phase 1 at 5 Hz (H_(7)_ = 9.904; *p* = 0.194; η^2^ = 0.087), but the analysis detected significant differences at 10 Hz (H_(7)_ = 14.834; *p* = 0.038; η^2^ = 0.122; Fig. 7*A*), although no specific difference among channels were detected by the *post hoc* analysis. For output B, Phase 2, the analysis detected significant differences for both emulated frequencies, 5 Hz (H_(7)_ = 15.394; *p* < 0.001; η^2^ = 0.08) and 10 Hz (H_(7)_ = 16.134; *p* < 0.001; η^2^ = 0.144; Fig. 7*B*). In both cases, no specific difference was detected by the *post hoc* analysis. In Phase 3, considering both outputs, the analysis detected significant differences at 5 Hz (H_(7)_ = 16.941; *p* = 0.018; η^2^ = 0.146) and at 10 Hz (H_(7)_ = 18.247; *p* = 0.011; η^2^ = 0.178; Fig. 7*C*).

The analysis detected significant differences for the transmission error in Phases 1, 2, and 3 depending on the number of cages used in each test (Fig. 7*D–F*). In Phase 1, the Kuskal–Wallis test indicated significant difference for both emulated frequencies, 5 Hz (H_(7)_ = 53.960; *p* < 0.001; η^2^ = 0.237) and 10 Hz (H_(7)_ = 52.764; *p* < 0.001; η^2^ = 0.244; Fig. 7*D*). For output B, Phase 2, the analysis detected significant differences for both emulated frequencies, 5 Hz (H_(7)_ = 57.369; *p* < 0.001; η^2^ = 0.380) and 10 Hz (H_(7)_ = 58.946; *p* < 0.001; η^2^ = 0.530; Fig. 7*E*). In Phase 3, considering both outputs, the analysis detected significant differences at 5 Hz (H_(7)_ = 57.369; *p* < 0.001; η^2^ = 0.405) and at 10 Hz (H_(7)_ = 58.946; *p* = 0.011; η^2^ = 0.406; Fig. 7*F*).

### *In vivo* validation

**Figure 8.**
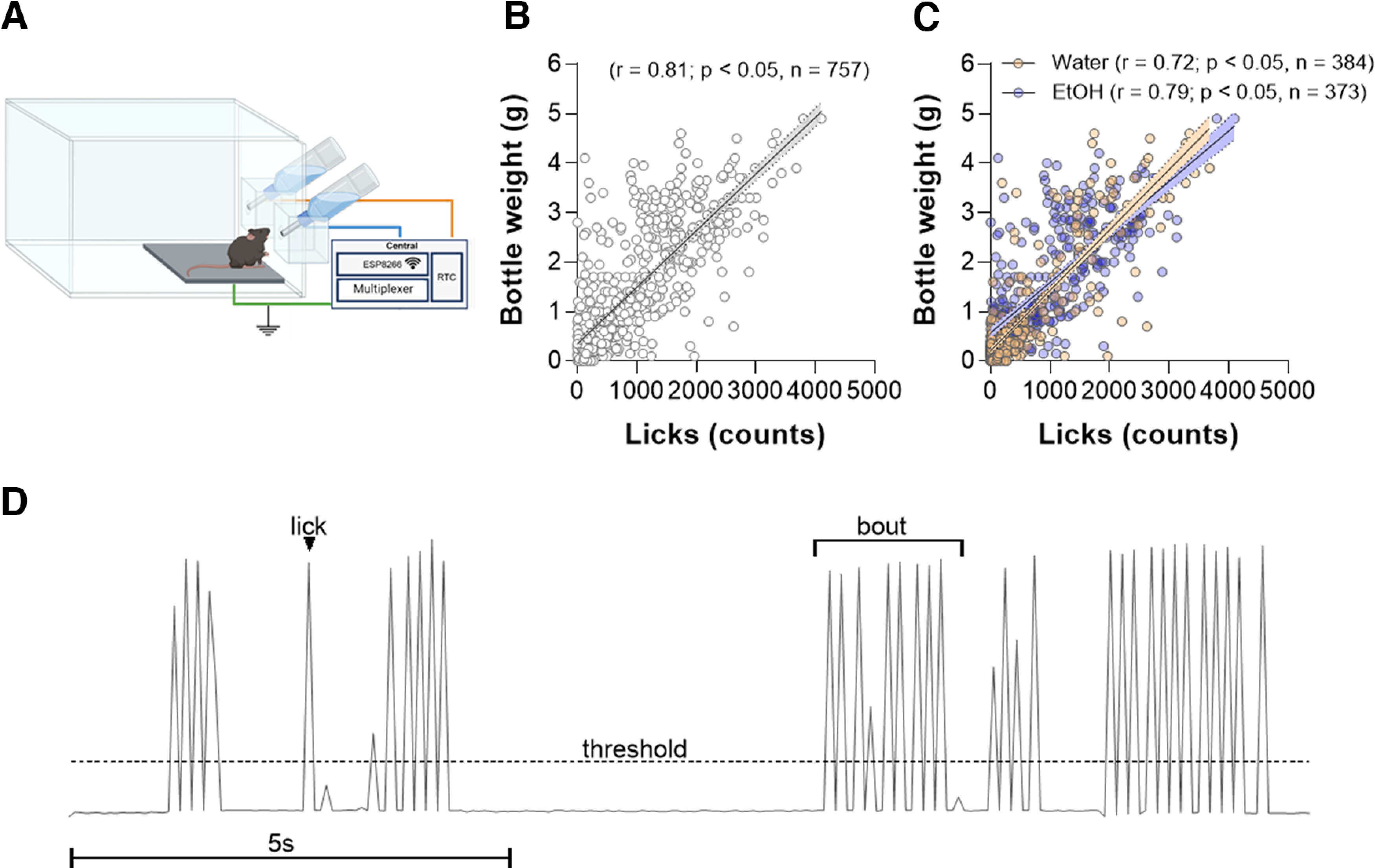
Results of the *in vivo* validation of the final lickometer system. ***A***, Schematic representation of the one final cage containing two lickometers coupled to the central unit. ***B***, Dispersion graph of the consumed liquid (difference of the bottle weight at the end of the experiment compared with the beginning of the experiment in grams - g) and total number of licks for each mouse in each session in the lickometer system. Spearman correlation revealed a strong correlation between the variables (*p* < 0.05). ***C***, Dispersion graph of the consumed liquid (g) and total number of licks for each mouse in each session in the lickometer system separated by different types of liquids: water and 10% ethanol. Correlation analysis revealed strong correlation independently of the liquid presented to the animals (*p* < 0.05). ***D***, Example trace of the raw acquired data demonstrating the detailed microstructure and timestamp licks that can be collected by our device. High peaks indicate licks events.

In the final validation step, we recorded the drinking behavior of 48 mice for 12 nights in three sets containing eight cages with two lickometers in each cage (Fig. 8*A*). A positive correlation (*r* = 0.81, *p* < 0.0001, linear regression fitness *R*^2^ = 0.72) was observed between the total amount of water or ethanol intake and lick counts acquired by our system. This correlation was also high if the type of liquid was considered (water: *r* = 0.72, *p* < 0.0001, linear regression fitness *R*^2^ = 0.73; ethanol: *r* = 0.79, *p* < 0.0001, linear regression fitness *R*^2^ = 0.59; Fig. 8*C*). In addition, Figure 8*D* shows an example trace of the acquired data, demonstrating the detailed microstructure and timestamps that can be collected by our device. Several microstructure parameters can be analyzed from the licks timestamps, for example: ILI, frequency, bouts, etc. in different time points of the experiments.

## Discussion

Although the complexity of drinking behavior is of key importance in different types of studies, most labs still measure only the total volume drank by animals, losing the detailed pattern of consumption. The microstructure can be easily measured with automatic lickometers (Barkley-Levenson and Crabbe, 2015; Cains et al., 2017; Frie and Khokhar, 2019; Godynyuk et al., 2019), but the commercially available ones are very expensive. In addition, the open-source options are designed for recording one animal per time. Thus, an extensive behavior protocol that demands several groups to be run together would cost a lot or demand great development efforts. We have developed and validated an automated lickometer device based on an open-source hardware platform with a user-friendly interface for monitoring measurements in real time. Among the advantages of our system, we highlight the extensive validation to show its reproducibility at an accessible low cost (the capital cost of one system, eight cages with two bottles each, was R$10,000.00, real, Brazilian currency, which represents about $2,000.00, in 2022). The device is easy to build and uses cheap components described in our GitHub webpage (https://github.com/kabrahao/BR_lickometer). Each house cage has two lickometers allowing researchers to offer two types of liquids to study preference and other variables. Each complete device is able to successfully collect data from eight animals at the same time, allowing complex multi-group behavioral neuroscience studies to be performed.

Three validation steps were conducted to reach the final prototype and to guarantee its reliability in detecting licking events. First, we verified the most appropriate sensor for this application, according to the existing prototypes in the literature, which are mostly based on photoelectric (Frie and Khokhar, 2019; Godynyuk et al., 2019) or capacitive sensors (Longley et al., 2017). It is important to point out that these previous studies did not compare the sensors or checked the influence of different variables on their data. The capacitive sensor responded with greater precision and stability when compared with the photoelectric sensor. The results were independent of animal age and sex, and the experience of reviewers did not have an effect on the results. The electronic principles behind the prototype components may explain the efficiency difference. In the case of the photoelectric sensor, when the light beam is interrupted by an object, the current stops and the Arduino counts as a lick. However, if the mouse stops in front of the photograph emitter interrupting the beam without licking the bottle, the Arduino still counts a lick. Thus, the photoelectric prototype measures only the interest in the liquid rather than the actual licking behavior. The photometer sensor was used by Frie and Khokhar (2019) and Godynyuk et al. (2019). On the other hand, the capacitive sensor works based on the electrical field between the 65-mm stainless steel nozzle and the ground platform. This system allowed the detection of a lick as a signal when the mouse touched the bottle nozzle with its tongue. Thus, our measurement is not only the interest in the liquid but the real licking behavior. It is important to point out that if the licking cabin is large, other parts of the mice’s body may touch the nozzle and a spurious lick may be counted. To avoid these false positives, we restricted the cabin to allow only the approach of the mice snout.

The translation of the system to wireless allowed us to better monitor eight animals simultaneously, with two capacitive sensors per cage. In order to evaluate the prototype performance, we proposed the *in silico* validation step with simulated licks at extreme and typical rates. This step was important to verify the reading and transmission errors from the proposed system. Ideally, we expected no differences between the simulated conditions and phases of validation in the same frequencies, since the hardware requirements between phases would be similar. However, some statistical differences were found among different phases and different frequencies. Despite some differences in specific situations, the reading error mean was lower than 0.1% and the transmission error mean was lower than 10% in all tests. Transmission error though was higher for some validation phases probably because of busier network bandwidth during the simulation experiment. Thus, for wireless transmission prototypes, it is important to verify whether other wireless equipment is turned off to avoid loss of information. Thus, errors tend to be even lower in regular situations, guaranteeing a good performance of the system.

In the third part of our study, we collected simultaneous data from 24 cages that recorded the interaction of each animal with two bottles, over 12 sessions. For validation, we correlated the total amount of liquid consumed, measured by weighting the bottle before and after the test, with the lick count, obtaining a positive correlation. Previous studies used validation methods similar to ours. In general, validation usually involves the exposure of experimental animals to voluntary liquid consumption protocols, allowing the correlation of the consumed volume with the number of licks registered (Raymond et al., 2018; Godynyuk et al., 2019; Renteria et al., 2020). A similar validation method was adopted by Longley et al. (2017), who have developed a device with capacitive sensors and analyzed data from animals exposed to an operating protocol to obtain sucrose or water under a progressive reinforcement scheme.

Our data demonstrate that the device is useful in assessing the microstructure of consumption in behavioral studies. Another important contribution of the proposed system is the versatility of bottle volumes that could be attached to the nozzle. To better accessibility of the mouse to the drinking cabin, we measured the best height and slope for the nozzle bottle to be ergonomic to the mouse features, considering cage and bottle support can influence licking behavior. Proper ergonomics reduce noise generated by the animal’s own body, such as paws or tails activating the sensor. The lickometer acrylic cage dimensions are ideal for mice or young rats, but it is also relevant to mention that it could be easily adapted for rats by changing the dimensions of the acrylic cage. Additionally, the removable wall that supports the drinking sippers could be changed to one with higher positioning of the sippers for adult rats, and the top metal grid could be changed to a higher set allowing rats to show normal rearing behavior.

Some expertise is desirable to reproduce the construction of the apparatus. First, basic electronics circuit knowledge is necessary to build the printed circuit board. Developing the final wireless prototype requires an intermediate programming background to design the firmware that sends the sensor data to the computer wirelessly and the user interface that receives data in real time. We believe that researchers will be able to build the device in three months or less with the expertise mentioned before.

There are limitations in our system. The device does not measure volume, so there is no real-time information about the exact amount of volume consumed in each lick or bout, for example. Furthermore, manufacturing our lickometer and installing the software requires knowledge beyond behavioral neuroscience, including software programming and hardware montage. Despite these limitations, the device is precise and valuable for drinking behavior measures at an accessible cost. In addition, the wireless adaptation allows the computer not to be necessary for the experimental room, reducing animal stress and changes in the light-dark cycle. In addition, the device has the potential to have data sent to the cloud, making it possible to monitor the experiment remotely and the potential to be coupled to *in vivo* neuroscience tools such as electrophysiological and photometrical neuronal recordings.

In conclusion, aiming to bring an innovative and low-cost solution for drinking behavioral studies, we proposed a lickometer system based on an open-source hardware platform with an user-friendly interface software, capable of simultaneously receiving data from eight automated cages with two drinking bottles each. Our results showed that the capacitive sensor presented appropriate recall and precision for this application. The device showed a reliable positive correlation between the total amount of liquid consumed and the licks count. Moreover, future work will involve adapting the system to send data to the cloud using the Internet network; algorithm improvements for creating an adaptive threshold; and system adaptation to include a volume sensor for automated measurement of consumed volume to enhance the study of licking microstructure.

## Synthesis

Reviewing Editor: Erica Glasper, The Ohio State University Decisions are customarily a result of the Reviewing Editor and the peer reviewers coming together and discussing their recommendations until a consensus is reached. When revisions are invited, a fact-based synthesis statement explaining their decision and outlining what is needed to prepare a revision will be listed below. The following reviewer(s) agreed to reveal their identity: Elizabeth Glover.

Synopsis

Rodent behavioral research has heavily relied on liquid intake assays for decades. Many fields appreciate measuring drinking behavior because it results in microstructural data. The authors of this manuscript describe a new, open-source instrument that will increase users ability to collect microstructural drinking-related data from rodents. Even though other do-it-yourself (DIY) presently exist, the unique design of this lick-o-meter has the potential to advance the field because it can produce more detailed licking behavior data, compared to manual assessment of liquid consumed. In this manuscript, a two-bottle arrangement in a single cage using off-the-shelf equipment is described. This is like a few other papers that use similar lick-o-meter designs; however, the main advancement in this manuscript is the use of wireless technology to centralize data collection on a single chip/processor as compared to having separate units for each cage/bottle pair. The validation methods used by the authors are valuable; they assure the potential user that the system accurately and precisely acquires microstructure data. While the resources provided in this manuscript have the potential to provide other laboratories with an affordable, well-validated DIY option for acquiring such data, the manuscript lacks important details that are required for the potential end user to build the devise themselves. Without this information, proper review of the methods cannot be truly performed. Below are the major and minor comments provided by the reviewers. Where appropriate, reviewers comments are directly quoted and can be found italicized font.

Major Comments

Methods

Methodological details are missing from this manuscript and may be obscured for anonymity (e.g., GitHub link). Since this information is not viewable, it is unclear whether the information provided within the link will be sufficient for another lab to build and implement the proposed apparatus. The information contained within the manuscript is not sufficient to build the apparatus. Specifically, methods describing how the entire apparatus is to be constructed, including the availability of parts and the labor required, are missing from the current manuscript. One reviewer raised significant concerns about availability and accessibility of supplies, stating a lot of Arduino and other small parts are replaced quickly by new models that are often incompatible with other parts. This can make the recipe for any given build obsolete within a relatively short period of time essentially requiring reinvention of the wheel (e.g., this is now the case for several DIY raspberry pi video recording set ups published in the last 5 years) . The authors should also estimate the amount of time, level of expertise, and necessary equipment that will be required to build the apparatus.

Definitions within the manuscript are unclear.

Please redefine precision and recall; definitions should in include terms of true/false positives and true/false negatives . The definition of relevant instance is unclear. Is this a true positive? What does it mean for a relevant instance to be retrieved? Does that mean that the computer noted it?

It appears that a separate cage for acquiring drinking data was designed in Figure 3B but this is not mentioned within the methods section or elsewhere. This methodological difference deviates from field standards, where rodents remain undisturbed in their home cages. As the manuscript currently reads, it appears that rodents are relocated to a specialized cage for ∼16h/day. The authors are encouraged to address these methodological differences (if they indeed exist) more clearly and discuss this as a possible limitation in the design.

The authors need to provide information on how video lick analysis was performed.

Specifically, 1) are viewers hand-coding millisecond resolution licks and 2) how is this accurately distinguished from close approach or non-lick touches to the sipper ?

The statistics described by the authors (e.g., t-test) are not in line with the data that are being provided. The following questions should be addressed: Are the authors comparing the average of multiple sessions for each animal for each lickometer type or is each session being treated as an individual data point? If the authors have used the latter, then a nested analysis is more appropriate. The presentation individual data is very valuable; however, inappropriate presentation can lead to misinterpretation or misunderstanding. The authors are encouraged to graph the data to best reflect the analysis that was performed; this will not preclude the display of individual data points.

Figures

While Figure 3 C is helpful, the authors are encouraged to include a detailed birds eye photo of the three different setups . This should include actual photographs that include dimensions. Figure 4 requires clarity. Neither the image nor the legend describes what the bars in the graphs represent. Is each bar a single prototype? If so, what do the individual data points represent? Are those individual trials? Without the inclusion of these data, it is not possible to evaluate the appropriateness of the statistics.

Also, please clarify whether the data in Figure 4D are the same as Figures 4B and 4C but are just displayed differently. Figure 6 would benefit from additional information. The authors suggest that this new, DIY device will be good for obtaining data on drinking patterns and time stamped licking data but there isn t evidence of this in the present manuscript. It is suggested that the following be added to the current manuscript: session averages for total fluid consumption (mL) and g/kg ethanol, relationship between g/kg ethanol and lick number, cumulative lick records (and discussion of how these values compare to those in existing literature) .

Discussion

The authors should discuss the error rates of their new, DIY system and other systems currently available. While other DIY systems may not have this information readily available, commercial systems should be able to produce validation results.

The authors should discuss whether this system is for exclusive use in mice or whether it is compatible in other model systems, like rats.

Minor Comments

Introduction

Line 60: The authors cite Gero et al., 2019, however, this reference does not involve consumption of alcohol.

Line 61: The microstructural changes reported in the Darevsky papers are observed in a model of compulsive-like drinking but not necessarily indicative of this phenotype. Please provide evidence from the literature that equates compulsive drinking to drinking pattern reported by Darevsky.

Methods

Lines 135-136: The following statement should be expanded: Researchers observed if the dimension and position of the drinking bottle were ergonomic for mice. Please define what it means to be ergonomic for the mouse. Include description of the angle of sipper, height of sipper, etc. . Ergonomics were again discussed on lines 426-432, but details are not provided to evaluate the usefulness of this paragraph.

Interlick interval (ILI) is typically reported as the number of licks per second rather than acquisition rates (Hz). Upon first mention (Line 188), please convert Hz to ILI so that behaviorists have perspective on what ILI this data acquisition rate can resolve . Please revise the incomplete sentence on Line 247.

Results

Lines 260-262: Figure 2 is referred to, but the authors may have been referring to Figure 4. Please clarify.

Discussion

Line 380: Please add the missing parentheticals on reference.

Line 399: The authors should include a discussion of the design of the cabin; photos that include the measurements should be provided. Figures and Figure Legends Figure legends should include the mention of the statistical tests used, including p-values). This is necessary for all figures with significance group differences. More information is needed in Figure 1:

Show also Arduino with SD, Real time clock and LCD plugged in.

Show wiring diagram with Arduino, all components and photos of actual device.

Figure 2: Please define what the numerals and letters signify. For example, do they refer to cages and bottles?

Figure 5: Greater detail is needed regarding the box plots in Figure 5. For example, please describe what the colors represent, if the lines indicate mean or quartile and what the individual data points represent.

Discussion

The authors are encouraged to discuss the lack of high bandwidth WIFI in many vivaria. The authors should discuss the ease with which this system can be converted to a wired ethernet system to maintain cloud-based capture of data .
