# Supplementary figures and images for: Development of Eight Wireless Automated Cages System with Two Lickometers Each for Rodents
Source: eNeuro. 2022 Aug 3;9(4):ENEURO.0526-21.2022. doi: 10.1523/ENEURO.0526-21.2022 (PMC9355285; doi:10.1523/ENEURO.0526-21.2022)

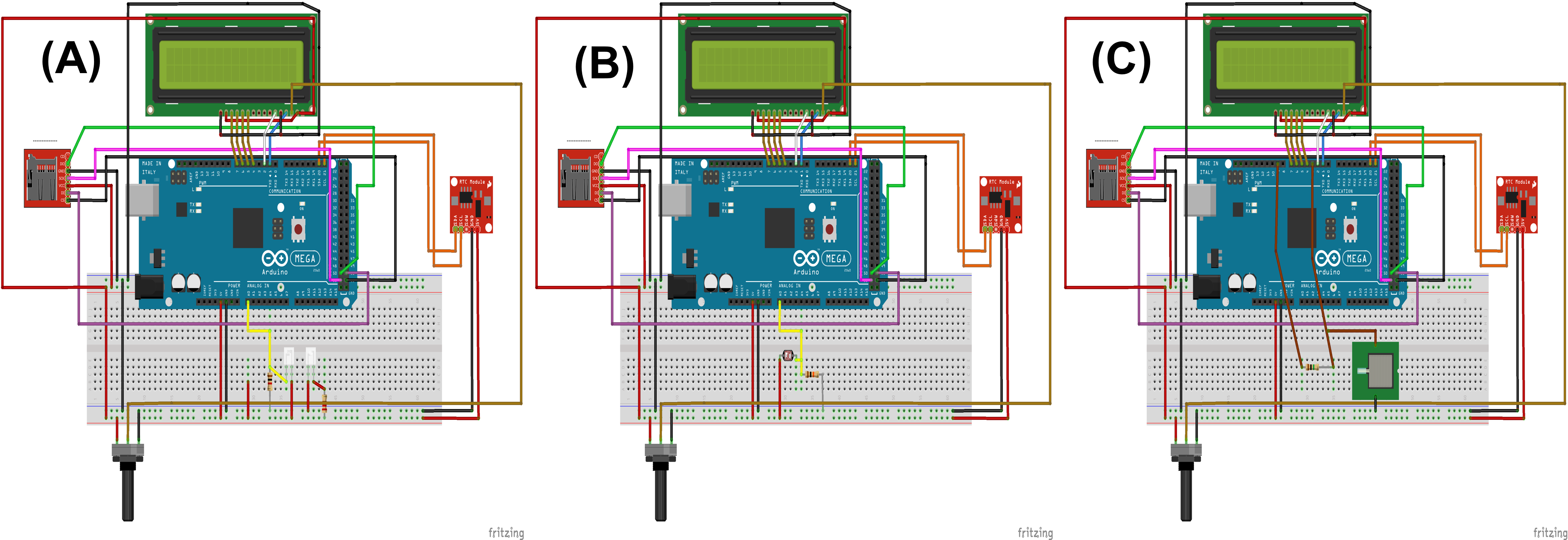

Supplement: Extended DATAFigure 1-1 — Extended figure shows the electronic schematic diagram of the three built prototypes: (A) photoelectric, (B) LDR, and (C) capacitive sensor. Download Figure 1-1, TIF file. [file enu-eN-MNT-0526-21-s02.tif]
